# O-GlcNAcylation: A Nutrient-Sensitive Metabolic Rheostat in Antiviral Immunity and Viral Pathogenesis

**DOI:** 10.3390/cells14211743

**Published:** 2025-11-06

**Authors:** Thomas I. Odo, Maya Saleh

**Affiliations:** Centre Armand-Frappier Santé Biotechnologie, Institut National de la Recherche Scientifique (INRS), Laval, QC H7V 1B7, Canada; thomas-ikechukwu.odo@inrs.ca

**Keywords:** virus, infection, innate immunity, host–pathogen interaction, inflammation, pattern-recognition receptors, immunometabolism, nutrient sensing, glycosylation, post-translational modification

## Abstract

Viruses account for the most abundant biological entities in the biosphere and can be either symbiotic or pathogenic. While pathogenic viruses have developed strategies to evade immunity, the host immune system has evolved overlapping and redundant defenses to sense and fight viral infections. Nutrition and metabolic needs sculpt viral–host interactions and determine the course and outcomes of the infection. In this review, we focus on the hexosamine biosynthesis pathway (HBP), a nutrient-sensing pathway that controls immune responses and host–viral interactions. The HBP converges on O-GlcNAcylation, a dynamic post-translational modification of cellular proteins, that emerged as a critical effector of immune cell development, differentiation, and effector functions. We present a broad overview of uncovered O-GlcNAc substrates identified in the context of viral infections and with a functional impact on antiviral immunity and viral restriction, or conversely on exacerbating viral-induced pathologic inflammation or viral oncogenesis. We discuss the clinical implications of these findings, current limitations, and future perspectives to harness this pathway for therapeutic purposes.

## 1. Introduction

Viruses account for the most abundant biological entities in the biosphere [1]. They can be either symbiotic or pathogenic [1,2]. Viral pandemics have informed our understanding of viral emergence, transmission, and host–pathogen interactions [3]. Coevolutionary selective pressures have shaped both viral evasion and host defense mechanisms [4,5]. Viruses evade immune recognition through a plethora of sophisticated strategies, including modification of their molecular patterns to avoid recognition by the innate immune system, proteolytic cleavage of host innate sensors, inhibition of antigen presentation, subversion of apoptosis and autophagy, rapid antigenic variation, and cytokine mimicry [6,7,8]. The host immune system has also evolved overlapping and redundant defenses to fight viral infections. Cells of the innate immune system orchestrate immediate defenses through a plethora of constitutive and inducible mechanisms (Box 1). Multiple innate sensors detect the same or overlapping viral molecular patterns, providing backup if one is compromised (reviewed in [5]), and converge on the induction of type I, II, and III interferons, and of interferon-inducible genes, to induce an antiviral state [9,10]. The adaptive immune system bolsters the innate antiviral response with antibodies that neutralize extracellular viruses and cytotoxic T cells that eliminate infected cells, to establish specific long-term immune memory [11]. This reciprocal adaptation plays out across scales, from molecular interactions between viral epitopes and host receptors to population-level phenomena like antigenic drift. Recent theoretical and computational models illustrate how viral populations move through antigenic space in response to immune pressure [4]. Virus–immune coevolution is not merely a conceptual framework but a quantifiable process with implications for understanding viral persistence, vaccine design, and the limits of immune memory [12].

Box 1 **Mechanisms of innate immune cells in anti-viral defense.**

Innate immunity provides first-line defenses against viral infection. Through constitutive and inducible mechanisms, mediated by immune and barrier cells, it restricts viral replication and shapes anti-viral adaptive immunity [13].

- Epithelial and endothelial cells are barrier cells that detect viral patterns via specialized sensors and respond by secreting type I/III IFNs, chemokines, and antimicrobial peptides. They also coordinate immune cell recruitment through the upregulation of adhesion molecules and cytokines [14,15].
- Natural killer (NK) cells are cytotoxic innate lymphocytes that scan for virally infected cells lacking MHC I expression or displaying stress-induced ligands. Upon activation, they kill target cells via perforin and granzyme release and secrete IFN-γ to enhance macrophage and dendritic cell function [16].
- Plasmacytoid dendritic cells (pDCs) are key amplifiers of the antiviral state as they are principal producers of type I interferons (IFNs), a class of cytokines critical for antiviral defense [17].
- Conventional dendritic cells (cDCs), particularly cDC1, cross-present viral antigens to prime CD8^+^ T responses and secrete cytokines such as IL-12 that polarize downstream T cell responses and serve as link between innate and adaptive immunity [18,19].
- Macrophages and monocytes are phagocytic sentinels that engulf viral particles, secrete pro-inflammatory cytokines (e.g., TNF-α, IL-1β), and present antigens to adaptive cells. Depending on the virus, however, these cells may also serve as sites of replication or contributors to immunopathology [20,21].
- Granulocytes are increasingly recognized for their contributions to antiviral immunity. Their presence has been documented at respiratory mucosal barriers, particularly during early inflammatory responses to respiratory viral infections, including respiratory syncytial virus (RSV), influenza, and SARS-CoV-2 [21,22]. While these granulocytes can facilitate viral clearance through direct and indirect effector functions, their excessive or dysregulated activation has also been implicated in tissue damage and exacerbated inflammation, particularly in severe respiratory infections such as influenza and COVID-19 [23,24].
  ○ Neutrophils, the most abundant circulating leukocytes, are rapidly recruited to infection sites, where, in addition to degranulation and production of reactive oxygen species (ROS), they form neutrophil extracellular traps (NETs), which immobilize and neutralize viral particles [24].
  ○ Eosinophils degranulate to release cytotoxic mediators, secrete ribonucleases (RNases) capable of degrading viral RNA, and modulate the recruitment and activation of other immune cells [25].
  ○ Mast cells release autocoids, proteases, and interferons, which increase vascular permeability, recruit immune cells, and restrict viral replication [26].

Nutrition and metabolic needs sculpt viral–host interactions and determine the course and outcomes of the infection [27,28]. Viruses, as obligate intracellular parasites, are entirely dependent on host-derived nutrients for energy and biosynthetic precursors, including nucleotides, amino acids, and lipids, to support replication, virion assembly, and spread. Similarly, the host immune system undergoes rapid and dynamic metabolic reprogramming during infection to support cellular proliferation, cytokine production, and effector functions. These opposing demands place cellular metabolism at the center of host–pathogen interactions [29]. Nutrient sensing has emerged as a critical regulatory mechanism by which immune cells detect fluctuations in nutrient availability and coordinate metabolic responses to maintain homeostasis and achieve effective responses to infection [30]. Undernutrition impairs innate and adaptive immunity, whereas overnutrition, particularly obesity, induces chronic low-grade inflammation and predisposes individuals to more severe viral disease outcomes [31,32]. Mechanistically, this relationship is controlled by the overlap of nutrient and pathogen-sensing pathways. Pattern recognition receptors (PRR), mammalian target of rapamycin (mTOR), AMP-activated protein kinase (AMPK), the hexosamine biosynthesis pathway (HBP), and O-GlcNAcylation are not only responsive to nutrient fluxes and metabolic stress but also to infection and danger signals [33].

In this overview of the recent literature, we present a comprehensive discussion of identified O-GlcNAcylation substrates involved in host–viral interaction, precise their O-GlcNAcylation sites, overlapping PTMs, and the functional impact of their modification on viral control or pathogenesis. We further outline clinical studies targeting this pathway. Lastly, we discuss current limitations of O-GlcNAcylation research, outstanding questions, and future directions towards rational clinical application of metabolic inhibitors targeting this pathway to treat viral infections.

## 2. The Hexosamine Biosynthesis Pathway and O-GlcNAcylation: From Nutrient Sensing to the Control of Immunity

The HBP has emerged as a key pathway in immune regulation with the capacity to intercept signals from both extracellular nutrients and intracellular metabolic intermediates (Box 2).

Box 2 **Immunometabolic pathways underpinning antiviral immunity.**

Metabolic reprogramming is a defining feature of immune activation and viral defense. Upon sensing infection, immune cells remodel core metabolic pathways to meet the heightened energetic and biosynthetic demands of proliferation, cytokine production, and effector function [31].

- **Glycolysis:** Glycolysis converts glucose into pyruvate, generating ATP and metabolic intermediates needed for biosynthesis. It supports rapid energy production independent of oxygen through lactate formation. In immune cells, glycolysis fuels activation, proliferation, and effector cytokine synthesis during infection or inflammation [29,34].
- **TCA Cycle:** The tricarboxylic acid (TCA) cycle oxidizes acetyl-CoA derived from pyruvate or fatty acids to generate NADH and FADH_2_, which fuel oxidative phosphorylation for ATP production. It also metabolizes amino acids such as glutamate. The cycle yields intermediates including citrate, α-ketoglutarate, and succinate that serve as biosynthetic precursors and metabolic signals regulating gene expression and immune activation [35]. In immune cells, the TCA cycle supports oxidative metabolism that underlies quiescence, memory formation, and the resolution of inflammation [29].
- **Pentose Phosphate Pathway (PPP):** The PPP branches from glycolysis to generate NADPH and ribose-5-phosphate. NADPH supports lipid synthesis and antioxidant defense, while ribose-5-phosphate enables nucleotide production, including uridine triphosphate (UTP) [29]. This pathway sustains redox balance and biosynthetic processes during immune activation [36].
- **Lipid Metabolism:** Lipid metabolism includes anabolic fatty-acid synthesis and catabolic fatty-acid oxidation. Synthesis provides membrane lipids and signaling molecules, whereas oxidation generates ATP and acetyl-CoA [37]. The balance between these opposing arms determines inflammatory versus regulatory immune phenotypes [29].
- **Hexosamine Biosynthesis Pathway (HBP):** The HBP integrates glucose, amino acid, fatty acid, and nucleotide metabolism to produce UDP-GlcNAc. This metabolite serves as the donor substrate for protein O-GlcNAcylation, a nutrient-sensitive post-translational modification. Through O-GlcNAcylation, the HBP links metabolic flux to immune signaling and antiviral responses [29,38].

**Figure 1:**
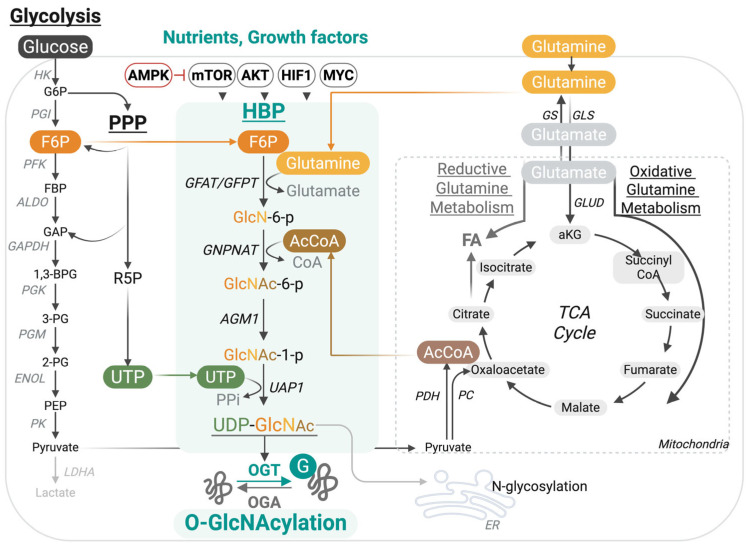
The hexosamine biosynthesis pathway (HBP) links nutrient availability to dynamic protein O-GlcNAcylation. The HBP integrates inputs from multiple nutrient and metabolic sources to generate UDP-GlcNAc, the donor substrate for protein O-GlcNAcylation. A fraction of glucose entering glycolysis is diverted at fructose-6-phosphate (F6P), which, together with glutamine, fuels GFAT/GFPT, the rate-limiting step of the pathway. Additional inputs are provided by acetyl-CoA from the tricarboxylic acid (TCA) cycle, uridine triphosphate (UTP) from pyrimidine biosynthesis, and nutrient-sensitive regulators (AMPK, mTOR, AKT, HIF1, MYC). The resulting UDP-GlcNAc is utilized by O-GlcNAc transferase (OGT) to modify serine/threonine residues on nuclear, cytoplasmic, and mitochondrial proteins, while O-GlcNAcase (OGA) removes the modification.

Key substrates driving the HBP include glucose, glucosamine, amino acids, fatty acids, and nucleotides (Figure 1). The HBP consumes 2–3% of glucose from the glycolytic pathway in the form of fructose-6-phosphate (F6P). Similarly, it derives acetyl-CoA (AcCoA) from the oxidation of pyruvate, fatty acids, and amino acids in the tricarboxylic acid (TCA) cycle, and obtains uridine triphosphate (UTP) from the de novo pyrimidine biosynthesis pathway. Exogenous glucosamine or GlcNAc can also be salvaged into the pathway by conversion to GlcNAc-6-phosphate (GlcNAc-6-p), bypassing the HBP rate-limiting step at Glutamine-fructose-6-phosphate amidotransferase (GFAT/GFPT) [39]. HBP links nutrient sensing to a dynamic and reversible post-translational modification (PTM) termed O-GlcNAcylation. This PTM of cytoplasmic, mitochondrial, and nuclear proteins regulates protein–protein interactions, protein stability, and DNA-binding affinity, often in synergy or competition with other PTMs, such as phosphorylation and ubiquitination. It is catalyzed by one writer, O-GlcNAc transferase (OGT), that couples UDP-GlcNAc to Serine (S) or Threonine (T) residues in target substrates and is reversed by an eraser termed O-GlcNAcase (OGA) [39,40]. Emerging research demonstrates the indispensable role of this PTM in immunity, from controlling immune cell development and differentiation to fine-tuning inducible innate and adaptive immune responses, through direct modification of central effectors (Table 1).

In T cells, O-GlcNAcylation peaks shortly after T cell receptor (TCR) engagement and both NFAT and NF-κB interact with OGT and are O-GlcNAcylated, which promotes IL-2 production and T cell activation [52,53,54]. The precise role of O-GlcNAcylation in T cell development was investigated by Swamy et al., who demonstrated that O-GlcNAcylation is central in driving T cell self-renewal and transformation [58]. Using different T cell-specific *Ogt* knockout mouse strains, they showed that Ogt is required for the rapid self-renewal of T cell progenitors, malignant transformation of Pten-deficient thymocytes, and T cell positive selection and maturation. They demonstrated that c-Myc is O-GlcNAcylated, which enhanced its protein stability, and that conversely, c-Myc drives O-GlcNAcylation by upregulating nutrient uptake [58]. O-GlcNAcylation is equally essential for B cell development, survival, and antibody responses. Wu et al. revealed the role of Lyn S19 O-GlcNAcylation in promoting Syk recruitment for efficient B cell receptor (BCR) signaling [60]. Lee et al. further highlighted the importance of c-Myc O-GlcNAcylation in pre-B cell proliferation [59]. More recently, Jeon et al. showed that O-GlcNAcylation regulates chromatin architecture at the immunoglobulin locus, impacting V(D)J recombination during early B-cell development [61]. Using genetic and pharmacological approaches, they showed that caloric restriction, ketogenic diets, or OGT knockdown or inhibition led to a biased defect in V(D)J recombination and a selective reduction in distal VH gene usage. In contrast, Oga inhibition with Thiamet G, which boosts O-GlcNAcylation, rescued VH gene recombination. O-GlcNAcylation was shown to regulate the cohesin complex, essential for IgH loop extrusion, through direct modification of several of its components, including YY1, CTCF, SMC1, and SMC3. Notably, it promoted SMC1 and SMC3 interaction, as well as YY1 and CTCF DNA-binding to the IgH locus. Collectively, these results link nutritional status and O-GlcNAc metabolism to antibody repertoire diversity.

Besides lymphocyte development, O-GlcNAcylation regulates T cell lineage commitment and effector functions. The lineage stability and suppressive program of regulatory T cells (Treg) is governed by Ogt that controls both FoxP3 stability, by preventing its K48 ubiquitin-mediated degradation, and STAT5 activation [56]. The central role of Ogt in this compartment was demonstrated by the severe scurfy-like autoimmune disease observed in mice harboring Treg-specific loss of *Ogt* [56]. In Th17 cells, O-GlcNAcylation supports the RORγt transcriptional program. This is mediated by acetyl-CoA carboxylase 1 (ACC1) modification on S966 and S967 that boosts its enzymatic activity, which contributes to enhanced production of RORγt ligands [57]. It is thus posited that the chronic inflammation elicited in metabolic diseases might be driven by aberrant O-GlcNAcylation, leading to heightened ACC1 activity and pro-inflammatory Th17 responses.

While the role of O-GlcNAcylation in myeloid cell development has not been elucidated, key effectors of macrophage metabolism and inflammatory signaling, e.g., RIPK3 [41], S6K1 [42], and IRF5 [43], are O-GlcNAcylated with context-dependent functional outcomes on macrophage inflammatory responses. For instance, while the O-GlcNAcylation of RIPK3 [41] and S6K1 [42] attenuates their activity in inflammatory signaling, that of IRF5 [43] exerts the opposite effect and leads to heightened inflammation in viral infection, as we describe below.

## 3. O-GlcNAc Modulation of Host Antiviral Innate Immunity

**Figure 2:**
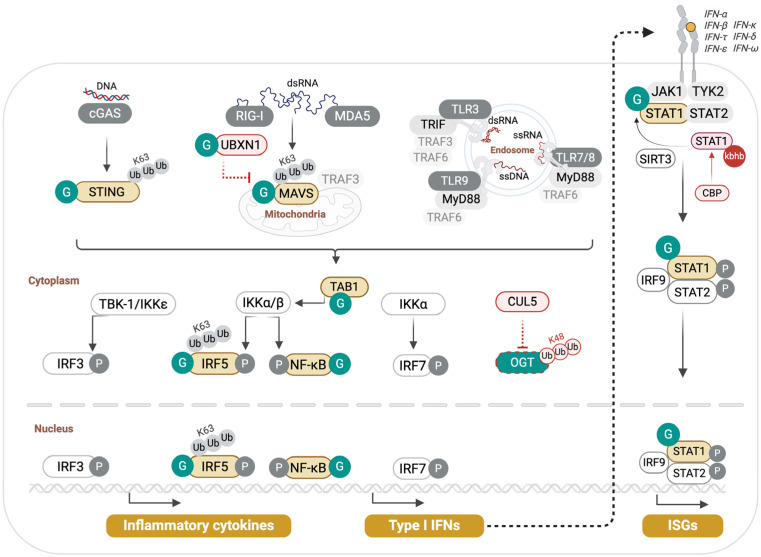
O-GlcNAcylation in innate antiviral signaling. Pattern recognition receptors (PRRs) such as cGAS, RIG-I, MDA5, and TLRs detect viral nucleic acids and trigger downstream signaling through STING, MAVS, and adaptor complexes that converge on IRF3/5/7 and NF-κB to drive type I interferon and pro-inflammatory cytokine production. O-GlcNAc transferase (OGT) and the hexosamine biosynthesis pathway (HBP) integrate nutrient availability with these antiviral pathways by directly modifying central effectors. MAVS O-GlcNAcylation promotes K63-linked ubiquitination and antiviral signaling, while UBXN1 O-GlcNAcylation relieves its inhibitory effect on MAVS. STING requires O-GlcNAcylation for activation and IFN induction. IRF5 O-GlcNAcylation drives TRAF6-mediated ubiquitination and cytokine storm responses in severe influenza, whereas NF-κB and TAB1 O-GlcNAcylation promote inflammatory gene expression. STAT1 O-GlcNAcylation, which counteracts β-hydroxybutyrylation, is required for efficient IFNAR signaling. CUL5-mediated K48-linked ubiquitination mediates OGT degradation.

Among the early findings that demonstrated a central role of O-GlcNAcylation in promoting antiviral innate immunity is the identification of MAVS O-GlcNAcylation, shown to be required for efficient RIG-I-mediated restriction of RNA viruses (Figure 2).

Li et al. first observed that infection of macrophages with vesicular stomatitis virus (VSV) upregulated their glucose uptake and metabolism, leading to higher UDP-GlcNAc levels and total protein O-GlcNAcylation [44]. Myeloid-specific deletion of OGT (LysM-Cre x *Ogt^f/f^*, referred to thereafter as *Ogt^Δmye^*) resulted in impaired VSV-induced innate antiviral responses both in vitro and in vivo, leading to higher viral loads, heightened lung pathology, and mortality. S366 was identified as the O-GlcNAcylation site in MAVS critical for RLR Signaling. Indeed, while wild-type MAVS could rescue the phenotype of *Ogt*-deficient macrophages in activating IRF3 and NF-κB upon VSV infection, an S366A MAVS mutant failed to do so. O-GlcNAcylation of MAVS was shown to promote its TRIM31-mediated K63-linked ubiquitination, a PTM previously shown to mediate MAVS antiviral signaling [62]. Similar findings were reported by Song et al., who demonstrated that MAVS O-GlcNAcylation is essential for host defense against influenza A viruses (IAV) [45]. However, they identified multiple adjacent O-GlcNAc sites in MAVS (amino acids 324–347) that were collectively required for efficient MAVS signaling. Their results showed that glucosamine treatment of macrophages increased MAVS-O-GlcNAcylation and boosted antiviral signaling, and that glucosamine dietary supplementation significantly improved the survival of wild-type mice following infection with IAV or VSV, but not that of *Ogt^Δmye^*, *Mavs^−/−^*, or *Ifnar^−/−^* mice [45]. Glucosamine also protected neonatal mice from coxsackievirus A6 infection [45]. A further, albeit indirect, support of the Key role of MAVS O-GlcNAcylation in macrophage antiviral innate immunity came from Zhang et al., who identified a role of the E3 ubiquitin ligase Cullin5 (CUL5) in regulating OGT stability through K48 ubiquitin–proteasome degradation [63] (Figure 2). They demonstrated that myeloid-specific deletion of CUL5 (*Cul5^Δmye^*) augmented OGT levels, leading to enhanced MAVS O-GlcNAcylation and improved macrophage antiviral immunity. The authors demonstrated that OGT degradation by CUL5 contributed to respiratory virus-induced neutrophilic asthma exacerbations. Investigations of signals in the allergic microenvironment—that dampen antiviral immunity through OGT degradation—identified the epithelial alarmin thymic stromal lymphopoietin (TSLP) as a mediator of CUL5 gene induction.

At odds with the above three studies [44,45,63], Seo and colleagues reported that MAVS O-GlcNAcylation inhibited its function in antiviral signaling [46]. Using Sendai virus (SeV) as a model, they showed that O-GlcNAcylation declines during infection, partly due to host-mediated downregulation of OGT transcription. This reduction permits MAVS aggregation and interaction with TRAF3 to drive type I interferon production. Seo et al. identified a distinct O-GlcNAcylated site in MAVS (amino acids 249–257) whose removal enhanced antiviral signaling in epithelial cells. Collectively, these results illustrate the complexity and site-specific nature of O-GlcNAc-dependent modulation of MAVS function. While this PTM appears to bolster MAVS antiviral responses in macrophages, it leads to a contrasting effect in epithelial cells, as reported by Seo et al. [46]. Beyond differences in cell types, the discordant results might stem from differences in experimental conditions, viral models, and time of sampling.

Besides its direct modification, MAVS signaling is further controlled through the O-GlcNAcylation of its inhibitor UBXN1, which frees MAVS to interact with TRAF3 for signal transduction [47] (Figure 2). Xia and Jiang observed this by studying the role of p53 in antiviral innate immunity. They observed that deficiency in *p53* led to compromised antiviral responses to VSV in vitro and in vivo and demonstrated that p53 acted by boosting O-GlcNAcylation through transcriptional upregulation of the HBP rate-liming enzyme GFPT2 [47] (Figure 1).

O-GlcNAcylation is also a critical driver of STING-induced innate immunity to DNA viruses [48]. Li et al. showed that STING stimulation by poly(dA/dT) or herpes simplex virus type 1 (HSV-1) infection increased glycolysis, PPP, and HBP, leading to elevated serum UDP-GlcNAc and global O-GlcNAcylation. OGT knockdown reduced IRF3 phosphorylation and lowered secretion of IFN-β and IL-6 in HSV-1-infected cells and diminished transcription of multiple interferon-stimulated genes. O-GlcNAcylation of STING on T229 promoted its K63-linked ubiquitination. Cells expressing a STING-T229A mutant failed to activate TBK1, IRF3, and NF-κB, even when treated with the OGA inhibitor Thiamet G. Thus, O-GlcNAcylation at T229 is necessary for STING to transmit antiviral signaling (Figure 2).

The antiviral role of O-GlcNAcylation is bolstered by the positive regulation of the transcription factor STAT1 that orchestrates innate antiviral defenses [49]. Zuo et al. recently identified opposing effects of STAT1 β-hydroxybutyrylation and O-GlcNAcylation. They reported that β-hydroxybutyrylation of STAT1 increased with aging and led to attenuated induction of IFN-stimulated genes by decreasing STAT1–IFNAR2 interaction. STAT1 β-hydroxybutyrylation was shown to be mediated by cAMP response element-binding protein (CREB)-binding protein (CBP) and countered by the deacetylase Sirtuin 3 (SIRT3). STAT1 O-GlcNAcylation on T699 attenuated its β-hydroxybutyrylation by inhibiting CBP binding. Fructose supplementation boosted STAT1 O-GlcNAcylation and improved antiviral immunity in vivo. In *Sirt3*^−/−^ conditions, STAT1 β-hydroxybutyrylation was also diminished, indicating a cooperation between OGT and SIRT3 in enhancing STAT1 association with IFNAR2 and IFN-I signaling [49].

While O-GlcNAcylation promotes antiviral immunity, it can also lead to virus-induced inflammatory pathology, as demonstrated by Wang et al. in IAV infection [43]. O-GlcNAcylation of IRF5 on S430 was shown to enable its activation by TRAF6-mediated K63-linked ubiquitination. Glucosamine or OGA inhibition in IAV-infected wild-type mice elicited a lethal cytokine storm, whereas *Ogt^Δmye^* mice exhibited less body weight loss, better survival, and lower lung viral titers. Reconstitution of *Irf5^−/−^* BMDMs with wild-type IRF5 restored cytokine production, whereas the S430A mutant did not, even in the presence of the Oga inhibitor Thiamet G. These data show that the OGT–IRF5 axis, specifically IRF5 O-GlcNAcylation on S430, drives a cytokine storm in fulminant viral infections. Consistent with their experimental findings, Wang et al. showed that compared to healthy individuals, IAV-infected patients had higher serum glucose levels, higher PBMC global- and IRF5-specific O-GlcNAcylation, which correlate with their inflammatory status, in particular higher circulating IL-6 and IL-8 cytokine levels.

Collectively, a model emerges in which viral infection significantly boosts host glucose metabolism, which drives O-GlcNAcylation and positive regulation of antiviral innate immunity. However, in fulminant cases, O-GlcNAcylation can mediate virus-induced cytokine storm and inflammatory pathology, e.g., being mediated by excessive activation of IRF5. In individuals with type-2 inflammation, such as asthma, O-GlcNAc activation of STAT6 might also compromise frontline antiviral defenses, as STAT6 induces IL-25 that suppresses the production of type I and III interferons, as shown in rhinovirus infection [64], and through IL-33, can drive a “type-2 cytokine storm”, as observed in COVID-19 and influenza infections [65].

## 4. O-GlcNAc Interference with the Viral Machinery

In addition to regulating antiviral innate immunity, O-GlcNAcylation can directly interfere with the viral machinery (Table 2).

**Figure 3:**
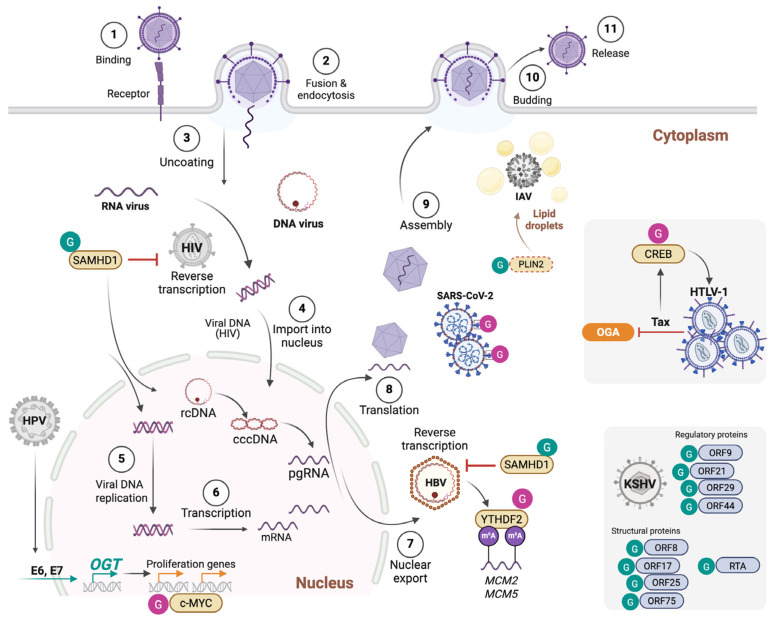
O-GlcNAcylation modulates multiple steps of the viral life cycle and viral oncogenesis. O-GlcNAcylation stabilizes the antiviral factor SAMHD1, thereby enhancing restriction of HIV-1 and HBV. In HBV infection, O-GlcNAcylation of the m^6^A reader YTHDF2 promotes stabilization and binding to MCM2/5 transcripts, supporting viral oncogenesis. In influenza A virus (IAV), OGT restricts replication by targeting lipid droplet metabolism via PLIN2 degradation, while in SARS-CoV-2, O-GlcNAcylation of the spike protein enhances stability and particle packaging. In HTLV-1, the viral oncoprotein Tax inhibits OGA, elevating CREB O-GlcNAcylation and driving proviral transcription. HPV E6/E7 stimulates OGT expression, leading to c-MYC O-GlcNAcylation and stabilization, which sustains cellular transformation. In KSHV, O-GlcNAcylation of multiple proteins inhibits viral replication and the latent-lytic cycle.

This has been demonstrated for human immunodeficiency virus (HIV-1), Kaposi’s sarcoma-associated herpesvirus (KSHV), hepatitis B virus (HBV), influenza A virus (IAV), and SARS-CoV-2. Jochmann et al. showed that glucosamine treatment or OGT overexpression inhibited HIV-1 transcription in infected primary CD4^+^ T cells. This was mediated by O-GlcNAcylation of the host transcription factor Sp1 and required functional Sp1-binding sites in the HIV-1 long terminal repeat (LTR) promoter [69]. While this evidence suggested a potential role of O-GlcNAcylation in restricting HIV-1 infection, the mechanism by which it occurred was not fully investigated, as the reported O-GlcNAcylation of Sp1 did not alter its nuclear levels or DNA-binding affinity to the LTR, suggesting that additional OGT substrates might be involved. In a different report, Jochmann et al. showed that OGT inhibits KSVH viral replication in a dose-dependent manner, potentially through direct modification of 18 viral proteins, demonstrated by mass spectrometry to be O-GlcNAcylated substrates [66] (Figure 3). Among these, ORF75 was confirmed to be O-GlcNAcylated in infected cells, and O-GlcNAc sites were identified in ORF3, ORF10, ORF8, ORF44, ORF21, and ORF29. Nearly half of the O-GlcNAcylated proteins are involved in DNA synthesis and replication, suggesting a central role for O-GlcNAc in regulating these processes, although this has not been formally demonstrated. Concordantly, Ko et al. showed that O-GlcNAcylation of KSVH replication and transcription activator (RTA [ORF50]) suppressed latent-lytic switch [67]. The HBP can additionally counteract viral replication through the O-GlcNAcylation of sterile alpha motif and histidine/aspartic acid domain-containing protein 1 (SAMHD1) [50], a nuclease and dNTP triphosphatase that restricts viral replication by cleaving viral genomes and depleting the dNTP pool. Hu et al. showed that inhibition or knockdown of GLUT1, GFPT1, or OGT increased HBV DNA replication. They identified that SAMHD1 is O-GlcNAcylated on S93, which protects it from K48 ubiquitin-mediated degradation and is required for its tetramerization and antiviral activity against HBV and HIV-1 [50] (Figure 3). In patients with chronic hepatitis B, UDP-GlcNAc levels, GLUT1 expression, and total O-GlcNAcylation are higher than in healthy controls, and SAMHD1 is O-GlcNAcylated in liver tissues. It is argued that enhancing O-GlcNAcylation might suppress HBV replication. In a recent report, Dong et al. identified a novel role of OGT in restricting IAV infection. Besides its effect on promoting MAVS antiviral immunity through MAVS O-GlcNAcylation, OGT exerted an additional antiviral function independently of its catalytic activity. The authors demonstrated that K908A catalytically inactive OGT dampened IAV infection both in vitro and in vivo. Mechanistically, OGT binds to IAV genomic RNA and limits lipid droplet accumulation, which is required for efficient IAV replication [70]. This was achieved by promoting perilipin-2 (PLIN2) K48-ubiquitination and proteosomal degradation (Figure 3). These findings place OGT at the junction of sugar and lipid metabolism pathways collectively engaged in the fight against IAV infection.

## 5. Viral Hijacking of the O-GlcNAc Pathway to Dampen Antiviral Defenses, or Enhance Infectivity or Viral Oncogenic Transformation

Fricke et al. demonstrated that respiratory syncytial virus (RSV) counters antiviral defenses by forming cytoplasmic inclusion bodies that sequester p38 and OGT, reducing stress-granule (SG) assembly and global O-GlcNAcylation that limit the infection [71]. Xu et al. described that O-GlcNAcylation of SARS-CoV-2 spike protein at S659 enhances infectivity by promoting more efficient pseudoparticle packaging [68] (Figure 3). They showed that OGT associates with spike protein and enhances its stability by antagonizing K48-ubiquitin tagging and proteosomal degradation. Using a four-plasmid system (pLenti6 luciferase, HIV Gag/pol, HIV Rev, and GFP-S) to produce SARS-CoV-2 pseudoparticles (SARS-CoV-2pp) with either wild-type or S659A spike, they showed reduced packaging efficiency and infectivity in pseudoparticles containing the S659A mutant spike protein. Their results suggest that while S659 O-GlcNAcylation does not affect ACE2 receptor binding, it is important for efficient pseudoviral assembly or packaging. The O-GlcNAc pathway, which is elevated in several cancers [72], is also exploited by some oncoviruses to drive tumorigenesis. In the case of human papillomavirus (HPV), Zeng et al. observed that inhibition of O-GlcNAcylation blunted HPV-driven tumorigenesis in vivo. They demonstrated that HPV E6 and E7 oncoproteins stimulated OGT expression, leading to heightened global O-GlcNAcylation and specific c-Myc O-GlcNAcylation on T58, which promotes its stabilization and drives cellular transformation [73] (Figure 3). Kim et al. explored the mechanism of HPV cervical tumor metastasis to the lung. They showed that OGT expression correlates with that of two factors known to contribute to viral oncogenesis and metastasis, namely Host Cell Factor 1 (HCF1) and C-X-C chemokine receptor type 4 (CXCR-4) in HPV-infected cells, but the mechanism linking OGT to their regulation was not fully elucidated [74]. In Human T-lymphotropic virus 1 (HTLV-1), Groussaud et al. showed that the viral oncoprotein Tax hijacks the cellular O-GlcNAc machinery by inhibiting OGA activity, which elevates CREB O-GlcNAcylation, promoting LTR activation [55] (Figure 3). This was validated by pharmacologic OGA inhibition or Tax overexpression that similarly increased CREB O-GlcNAcylation and LTR activity. CREB S40A mutation abolished Tax-induced O-GlcNAc modification of CREB and reduced Tax-mediated LTR activation, linking CREB O-GlcNAcylation to a proviral outcome. In hepatitis B virus (HBV)-associated hepatocellular carcinoma (HCC), Yang et al. showed that HBV upregulates the HBP, leading to increased global O-GlcNAcylation and specific modification of the N6-methyladenosine (m^6^A) reader YTH N6-Methyladenosine RNA Binding Protein F2 (YTHFD2) on S263 that drove tumorigenesis [51] (Figure 3). YTHDF2 protein levels were higher in HBV-HCC than in matched non-tumoral tissue, as well as in HBV-transgenic mouse livers. HBV extended YTHDF2 protein half-life, while OGT knockdown or S263A mutation shortened it through K48-linked ubiquitination and proteosomal degradation. YTHDF2 knockdown significantly impaired cell growth, colony formation, and motility in HBV-infected hepatoma lines, functions that were restored by wild-type but not the S263A YTHDF2 mutant. Mechanistically, YTHDF2 O-GlcNAcylation enhanced its binding to m^6^A-modified transcripts, including those of ‘Minichromosome Maintenance Complex’ components MCM2 and MCM5, that are vital regulators of DNA replication. Thus, HBV engages the OGT pathway to promote HCC tumor formation through YTHDF2 modification and induction of central proliferation effectors.

## 6. Harnessing the HBP-O-GlcNAc Pathway for Clinical Applications

(i) The HBP and O-GlcNAcylation as potential biomarkers of disease progression in viral infections. Severe influenza infection provides clinical evidence that O-GlcNAcylation correlates with disease severity [43]. Wang et al. noted that patients with acute influenza had significantly higher global O-GlcNAc levels and OGT expression in their PBMCs compared to healthy individuals. Heightened O-GlcNAc was linked to elevated serum cytokine levels in patients, linking O-GlcNAcylation to a cytokine storm phenotype in severe flu. Patients with chronic HBV infection also show higher UDP-GlcNAc levels and greater protein O-GlcNAcylation in their liver tissue compared to healthy controls [50]. In HBV-related HCC, the HBP-O-GlcNAc axis is often upregulated [51]. As mentioned before, YTHDF2 is heavily O-GlcNAcylated, and its levels correlate with worse HBV-HCC patient survival, implicating O-GlcNAcylated YTHDF2 as a potential biomarker of aggressive disease. Similarly, elevated O-GlcNAcylation is a marker of HPV-driven oncogenesis, e.g., in cervical cancer [73]. In clinical cervical tumor samples, HPV-positive lesions exhibit higher OGT and O-GlcNAc levels than HPV-negative tissue. The HPV16 E6–driven increase in total cellular O-GlcNAcylation is not only associated with tumorigenesis but also with disease severity, as higher OGT and O-GlcNAc levels correlate with advanced grade or malignant progression. Hence, O-GlcNAcylation can be measured as a clinically relevant biomarker of HPV-associated malignant transformation and progression.

(ii) HBP and O-GlcNAcylation as therapeutic targets in viral Infections: Several studies have underscored immunometabolic modulation as a feasible adjunct treatment in counteracting virus-induced hyperinflammatory states. For instance, in a retrospective study of hospitalized COVID-19 patients, Sukkar et al. reported that a eucaloric ketogenic diet was associated with reduced mortality, fewer ICU admissions, and a favorable trend toward lower IL-6 levels [75]. *N*-acetyl glucosamine (GlcNAc) that drives the HBP has been tested as a low-cost anti-inflammatory immunomodulatory agent for inflammatory joint illnesses, inflammatory bowel disease, and autoimmunity [76,77,78,79,80]. It has also been investigated as a potential anti-inflammatory treatment in respiratory viral infections. In a prospective observational study of COVID-19 patients, GlcNAc was associated with improved clinical outcome [81]. In addition, it has shown antiviral activity in vitro [82]. Beyond metabolic modulation, direct pharmacological enhancement of the O-GlcNAc pathway is now being evaluated clinically using OGA inhibitors. Ceperognastat (LY3372689) is the most clinically advanced OGA inhibitor [83]. Phase I trials demonstrated oral bioavailability, robust target engagement across dose ranges, >95% OGA enzyme occupancy, and an acceptable safety profile, supporting its progression to Phase II trials. Other OGA inhibitors have also advanced into clinical evaluation. ASN90 (ASN120290) is an orally bioavailable compound in Phase II development with early human studies demonstrating a dose-dependent increase in protein O-GlcNAcylation, reliable target engagement confirmed by positron emission tomography, and a favorable safety profile [84]. MK-8719 is another potent, selective OGA inhibitor that completed first-in-human testing [85]. Pharmacodynamic assays confirmed sustained elevation of O-GlcNAc in brain tissue, establishing the feasibility of systemic OGA inhibition in humans. Although these compounds were initially developed for tauopathies such as Alzheimer’s disease, their pharmacological properties establish OGA inhibition as clinically viable and highlight the potential for its repurposing in viral infections, where O-GlcNAcylation shapes host–viral interactions.

## 7. Conclusions

O-GlcNAcylation has emerged as a pivotal post-translational modification linking nutrient flux to immune and viral processes. By integrating signals from glucose, amino acids, lipids, and nucleotides through the hexosamine biosynthetic pathway, it functions as a nutrient-sensitive rheostat that regulates cellular metabolism and immune signaling. In the antiviral context, O-GlcNAcylation exerts multifaceted control of host defense, fine-tuning innate viral sensing pathways by directly targeting central effectors such as MAVS, STING, and IRF family members, while regulating adaptive responses through modulation of master transcription factors in T and B cell subpopulations [Table 1]. This balanced cycling of O-GlcNAc, regulated by the actions of OGT and OGA, ensures that immune activation remains metabolically sustained while preventing excessive inflammation and tissue injury. Through years of coevolution, viruses have evolved to exploit or subvert this modification to enhance replication, latency, and immune evasion [Figure 3]. The bidirectional relationship between O-GlcNAcylation and viral pathogenesis underscores its dualistic nature, being protective during acute infection but permissive to persistence or transformation under chronic metabolic stress.

Despite the growing literature, several fundamental questions remain unresolved. Firstly, how do OGT and OGA distinguish among thousands of potential substrates to generate context-dependent outcomes? The lack of a strict O-GlcNAcylation consensus sequence and the influence of subcellular localization together contribute to the complexity of deciphering this question. Secondly, from a spatiotemporal perspective, how do fluctuations in nutrient availability or infection stage reprogram the O-GlcNAc proteome across distinct immune cell compartments? Addressing this question will require the integration of advanced glycoproteomics, single-cell metabolomics, and high-resolution imaging approaches, particularly when coupled with emerging artificial intelligence tools capable of resolving these complex dynamics. Regarding pathway crosstalk, O-GlcNAcylation often interacts with phosphorylation, acetylation, and ubiquitination on shared residues, but whether these interactions represent antagonism, synergy, or sequential modulation during viral infection remains unclear. Furthermore, chronic metabolic disorders such as obesity and diabetes alter systemic HBP flux and global O-GlcNAc levels, yet their precise influence on antiviral immunity and viral persistence requires further exploration.

From a translational standpoint, the reversibility of O-GlcNAcylation offers an attractive therapeutic strategy. Pharmacological inhibitors or activators of OGA and OGT, such as Thiamet-G, MK-8719, or Ceperognastat, can modulate global O-GlcNAc levels, but their systemic application carries the risk of off-target effects due to the ubiquity of this modification. An open question is whether O-GlcNAcylation should be increased to strengthen antiviral signaling or decreased to stop viruses from exploiting host metabolism. Another major challenge is achieving cell-type-specific and temporally controlled modulation that enhances antiviral immunity without triggering hyperinflammatory responses or metabolic toxicity. Answering this will require integrative in vivo models that map O-GlcNAc fluxes across the immune system, infection stages, and tissue microenvironments, thereby paving the way for new possibilities in the emerging field of metabolic therapies.

Ultimately, O-GlcNAcylation serves as a central link between nutrient sensing and host–virus interaction. As analytical tools advance, answering the outstanding questions will bring the field closer to leveraging O-GlcNAcylation as both a biomarker and a therapeutic target for immune restoration in viral, inflammatory, and oncogenic diseases.

## Figures and Tables

**Table 1:** Select O-GlcNAcylated substrates in immunity or viral restriction.

Substrate	Identified Site	Effect on Substrate	Effect on Virus	Cell Type_Functional Impact	Reference
**RIPK3**	T467	Inhibition of phosphorylation	Proviral	Macrophages_Reduced necroptosis and inflammation	[41]
**S6K1**	S489	Inhibition of phosphorylation	Proviral	Macrophages_Reduced inflammation	[42]
**IRF5**	S430	Activation through K63-Ub	Antiviral	Macrophages, PBMCs, epithelial cells_Enhanced inflammation	[43]
**MAVS**	S366; domain 324–347	Activation through K63-Ub	Antiviral	Macrophages_Enhanced antiviral response	[44,45]
	S249, T250, T252, S253, S255, S256, S257	Inhibition	Proviral	Epithelial cells and fibroblasts_Reduced antiviral response	[46]
**UBXN1**	S75, T95, S132	Inhibition of UBXN1-MAVS interaction	Antiviral	Macrophages_Enhanced MAVS antiviral response	[47]
**STING**	T229	Activation through K63-Ub	Antiviral	Fibroblasts_Enhanced antiviral response	[48]
**STAT1**	T699	Activation by inhibition of kbhb	Antiviral	Fibroblasts_Enhanced antiviral response	[49]
**SAMHD1**	S93	Stabilization	Antiviral	Macrophages, Hepatocytes_Enhanced antiviral response	[50]
**YTHFD2**	S263	Stabilization	Proviral	Hepatocytes_Enhanced proliferation	[51]
**NFAT**	n.d.	Activation	Antiviral	T cell activation	[52]
**c-REL**	S350	Activation	Antiviral	T cell activation, FOXP3 suppression	[53,54]
**CREB**	S40	Activation	Proviral	HTLV-1 T cell_Enhanced viral transcription	[55]
**STAT5**	T38, S57, S58, S270, S273,	Activation by phosphorylation	Proviral	Treg_Enhanced suppressive program	[56]
**FOXP3**	T38, S57, S58, S270, S273	Stabilization	Proviral	Treg_Enhanced suppressive program	[56]
**ACC1**	S966, S967	Activation	Antiviral	Th17_Enhanced RORγt transcriptional program	[57]
**c-MYC**	T58	Stabilization	Antiviral	T cell, B cell_Enhanced proliferation	[58,59]
**LYN**	S19	Activation	Antiviral	B cell_Enhanced BCR signaling	[60]
**SMC1**	n.d.	Activation	Antiviral	B cell_VH gene recombination	[61]
**SMC3**	n.d.	Activation	Antiviral	B cell_VH gene recombination	[61]
**YY1**	T236	Activation	Antiviral	B cell_VH gene recombination	[61]
**CTCF**	T668	Activation	Antiviral	B cell_VH gene recombination	[61]

n.d., not determined; kbhb, lysine β-hydroxybutyrylation; Ub, ubiquitin; S, serine; T, threonine.

**Table 2:** Select O-GlcNAcylated viral substrates.

Virus	Substrate	Identified Site	Effect on Virus	Functional Impact	Reference
KSHV	ORF3	S278	Antiviral	O-GlcNAc transferase inhibits KSHV propagation and modifies replication-relevant viral proteins as detected by systematic O-GlcNAcylation analysis.	[66]
	ORF10	T225, T338, S594, T632, T709	Antiviral		
	ORF8	S92	Antiviral		
	ORF44	S727			
	ORF21	S62	Antiviral		
	ORF29	n.d.			
	ORF75	n.d.	Antiviral		
	RTA (ORF50)	T366, T367	Antiviral	O-GlcNAc suppresses transactivation and lytic reactivation	[67]
SARS-CoV-2	Spike	S659	Proviral	Spike stability and pseudoparticle packaging	[68]

n.d., not determined; ORF, open reading frame; RTA, replication and transactivation; S, serine; T, threonine.

## Data Availability

Not applicable.
